# Whole genome-based reclassification of several species of the genus *Microbispora*

**DOI:** 10.1371/journal.pone.0307299

**Published:** 2024-08-22

**Authors:** Noureddine Bouras, Ricardo A. R. Machado

**Affiliations:** 1 Laboratoire de Valorisation et Conservation des Ecosystèmes Arides (LVCEA), Faculté des Sciences de la Nature et de la Vie et Sciences de la Terre, Université de Ghardaia, Ghardaïa, Algeria; 2 Laboratoire de Biologie des Systèmes Microbiens (LBSM), Ecole Normale Supérieure de Kouba, Algiers, Algeria; 3 Experimental Biology Research Group, Institute of Biology, Faculty of Sciences, University of Neuchâtel, Neuchâtel, Switzerland; Chandigarh University, INDIA

## Abstract

The classification of *Microbispora*, a bacterial genus of significant ecological, agricultural, biotechnological, and clinical importance, has traditionally been carried out based on 16S rRNA gene sequences or phenotypic characteristics, which may lead to equivocal conclusions and it is not in line with the current standards. Moreover, some of the recent species descriptions have not been made using whole genome sequences (WGS), or when used, not all the species were included in the analyses. Consequently, some of the taxonomic conclusions drawn are equivocal, and therefore some currently accepted species should be synonymized. In this study, we revised the taxonomy of the genus *Microbispora* using digital DNA-DNA hybridization (dDDH) and average nucleotide identity (ANI) values, and by reconstructing phylogenetic relationships using whole genome sequences. Based on the clear phylogenomic separations and on the sequence divergence values, we propose to elevate *Microbispora rosea* subsp. *rosea* and *Microbispora rosea* subsp. *aerata* to the species level, and therefore to create *Microbispora aerata* sp. nov. with JCM 3076^T^ (= DSM 43176^T^ = ATCC 15448^T^ = IFO 14624^T^ = NBRC 14624^T^ = VKM Ac-1507^T^) as the type strain. Hence, with this proposition, the correct name for *Microbispora rosea* subsp. *rosea* is *M*. *rosea*. Furthermore, we propose to reclassify *M*. *camponoti* as a subspecies within *M*. *bryophytorum*. Consequently, we propose the creation of the following two subspecies: *Microbispora bryophytorum* subsp. *bryophytorum* subsp. nov. with DSM 46710^T^ (= CGMCC 4.7138^T^ = NEAU TX2-2^T^) as the type strain, and *Microbispora bryophytorum* subsp. *camponoti* subsp. nov., comb. nov. with DSM 2C-HV3^T^ (= DSM 100527^T^ = CGMCC 4.7281^T^) as the type strain. In addition, we propose to reinstate *M*. *amethystogenes* as an independent species and not as a *M*. *rosea* synonym, and reclassify “*M*. *cellulosiformans*” as a subspecies within *M*. *amethystogenes*. Hence, we propose the creation of the following two subspecies: *Microbispora amethystogenes* subsp. *amethystogenes* subsp. nov. with NBRC 101907^T^ (= DSM 43164^T^ = JCM 3021^T^ = NRRL B-2637^T^) as the type strain, and *Microbispora amethystogenes* subsp. *cellulosiformans* subsp. nov., comb. nov. with Gxj-6^T^ (= DSM 109712^T^ = CGMCC 4.7605^T^) as the type strain. Lastly, we propose *M*. *fusca* NEAU-HEGS1-5^T^ and “*M*. *tritici*” MT50^T^ as later homotypic synonyms of *M*. *triticiradicis* NEAU-HRDPA2-9^T^.

## Introduction

The genus *Microbispora* was initially described by Nonomura and Ohara [1] as a member of the family *Streptosporangiaceae* [2], and subsequently emended by Zhang et al. [3]. Most members of this genus exhibit slightly oval to cylindrical shapes with smooth surfaces, as evidenced by the presence of longitudinal paired spores on short sporophores branching from the aerial mycelium [4].

At the time of writing, the genus *Microbispora* comprises 13 species and two subspecies with validly published and correct names (https://lpsn.dsmz.de/genus/microbispora). These species are: *Microbispora bryophytorum* [4], *M*. *camponoti* [5], *M*. *catharanthi* [6], *M*. *clausenae* [7], *M*. *coralline* [8], *M*. *fusca* [9], *M*. *hainanensis* [10], *M*. *oryzae* [2], *M*. *siamensis* [11], *M*. *sitophila* [12], *M*. *soli* [13], *M*. *triticiradicis* [14], and *M*. *rosea*. *Microbispora rosea*, which is the type species, is divided into two subspecies: *M*. *rosea* subsp. *rosea* [1, 15] and *M*. *rosea* subsp. *aerata* [15, 16]. Three additional species were recently described, “*M*. *cellulosiformans*” [17], “*M*. *rhizosphaerae*” [18] and “*M*. *tritici*” [19], but their names were not yet validated. On the other hand, some species of *Microbispora* have been transferred to other genera. This includes *M*. *bispora* that was transferred to the genus *Thermobispora* [20], *M*. *ehinospora* that was transferred to the genus *Thermomonospora* [21], *M*. *mesophila* and *M*. *thailandensis* that were transferred to the genus *Sphaerimonospora* [22], and *M*. *vridis* that was declared homotypic synonym of *Actinomadura rugatobispora* [15].

The bacterial genus *Microbispora* comprises microorganisms of significant ecological, agricultural, biotechnological, and clinical importance. *Microbispora* species have been isolated from various ecological niches, including the cuticle of insects [5], rhizosphere soil [12], soil from cold regions [17], soil from hot springs [13], moss [4], plant roots [6, 14], and plant leaves [2]. These species play significant roles in ecological and agricultural contexts due to their capabilities in organic matter decomposition, soil structure improvement, and fertilization [23]. They are also effective as biocontrol agents [24], promote plant growth [25, 26], aid in the biodegradation of pollutants [27, 28], and contribute to waste treatment processes [23]. *Microbispora* species hold significant clinical potential due to their production of bioactive compounds. These include many antibiotics [18, 29, 30], antiviral agents [31], anticancer agents [32, 33], and neuroprotective agents [34].

The application of phylogenomic approaches has greatly contributed to the taxonomy of prokaryotic species. However, in some taxa, the classification is still based on the sequences of the 16S rRNA gene, or of house-keeping genes, and phenotypic characteristics, which may not be suitable according to current standards. With the emergence of high-throughput next-generation sequencing (NGS) technologies, a considerable number of *Actinomycetota* genomes are now accessible in public databases. This availability allows for comprehensive investigations into their evolutionary and taxonomic relationships. Phylogenetic methods that take into consideration full genome sequences as average nucleotide identity (ANI) and digital DNA-DNA hybridization (dDDH) have been developed, and are currently being validated and implemented in different bacterial groups with complex taxonomy. In spite of these advances, recent species descriptions in the genus *Microbispora* have lacked whole-genome sequencing (WGS), or, when used, not all species with validly and correct names have been included in the analysis. Consequently, this has led to erroneous taxonomic conclusions, and some species should be synonymized.

To address these taxonomic ambiguities, we used dDDH and ANI values, and constructed phylogenetic trees to revise the taxonomy of the genus *Microbispora*. Our analysis included all species with currently valid and correct names and several undescribed *Microbispora* strains with publicly available genome sequences. The dDDH and ANI values were compared with established cut-off values for bacterial species/subspecies delimitation. Our study based on whole-genome sequencing resolves the previously ambiguous taxonomy of *Microbispora*.

## Material and methods

### Genomic dataset

We conducted taxonomic analyses using genome data and employed various bioinformatics methods to assess the relationships among different species of *Microbispora*. The genome sequences of all *Microbispora* species and subspecies with valid and correct names, all described *Microbispora* species with non-validated names, and several undescribed *Microbispora* species were retrieved from the GenBank database (S1 Table). Additionally, some of species that were previously classified in the genus *Microbispora* were included in the analysis for comparison purposes. A summary of the characteristics of the genome sequences used in this study is presented in S1 Table. Genome completeness and contamination was assessed using CheckM (v1.2.2) [35].

### Phylogenetic relationship reconstructions

Whole genome-based phylogenetic trees were inferred using the Type (Strain) Genome Server (TYGS), a free bioinformatics platform available under https://tygs.dsmz.de [36, 37]. For the phylogenomic inference, all pairwise comparisons among the set of genomes were conducted using Genome BLAST Distance Phylogeny (GBDP) and accurate intergenomic distances inferred under the algorithm ’trimming’ and distance formula *d*_*5*_ [38], 100 distance replicates were calculated each. The resulting intergenomic distances were used to infer a balanced minimum evolution tree with branch support via FASTME 2.1.6.1 including SPR postprocessing. Branch support was inferred from 100 pseudo-bootstrap replicates each [39]. In addition, core genome-based phylogenetic trees were also constructed. To this end, genomes were first aligned using Roary 3.13.0 [40]. Genes to be considered core had to be present in 85% of the genomes with an 85% protein identity. Obtained alignments were used to build phylogenetic trees using FastTree 2.1.10 based on the Generalized Time Reversible Model (GTR). The trees were rooted using *Sphaerimonospora mesophila* NBRC 14179^T^ as the outgroup. The National Center for Biotechnology Information (NCBI) accession numbers of the sequences used for these analyses are shown (S1 Table). 16S rRNA-based trees were inferred by using the maximum-likelihood method based on the Tamura-Nei model (S1 and S2 Figs). The tree with the highest log likelihood is shown. The percentage of trees in which the associated taxa clustered together is shown next to the branches. The tree is drawn to scale, with branch lengths measured in the number of substitutions per site. There were a total of 1349 positions in the final dataset. Evolutionary analyses were conducted in MEGA7 based on 100 replications. 16S rRNA gene sequences were obtained directly from the whole genome sequences using the bacterial ribosomal RNA predictor Barrnap 0.9 [41], or retrieved from the NCBI databank using the accession numbers provided in the original publications or from the repositories where the type strains were deposited. High sequence similarities were observed independently of the origin of the sequences (S1 and S2 Figs). Graphical representation and edition of the phylogenetic trees were performed with Interactive Tree of Life (v3.5.1) as indicated by Chevenet el al. [42] and Letunic and Bork [43].

### Sequences similarity values

All pairwise comparisons among the set of genomes were conducted using GBDP and accurate intergenomic distances inferred under the algorithm ’trimming’ and distance formula *d*_*5*_ [38], 100 distance replicates were calculated each. Digital DNA-DNA hybridization (dDDH) values and confidence intervals were calculated using the recommended settings of the GGDC 4.0 [37, 38]. Non-symmetrical average nucleotide identity (ANI) values were calculated using FastANI [44]. To delimit species and subspecies using dDDH values, the thresholds of 70 and 79%, respectively, were considered [45]. To delimit species using ANI value, the threshold of 95–96% is considered [44].

## Results and discussion

### 16S rRNA gene based phylogenetic analyses and sequence comparisons

Pairwise sequence comparisons of almost complete 16S rRNA gene sequences of all *Microbispora* child taxa, including species with valid and correct names, synonymized species, and species with not yet validated names (S2 Table), revealed a high degree of genetic similarity across them (S2 Fig). Consistently, phylogenetic trees reconstructed using these sequences show that this gene marker provides little resolution to effectively differentiate various taxa (S2 Fig). In addition, the phylogenetic tree revealed a high degree of phylogenetic relatedness between the type strain of certain species such as *M*. *camponoti* and *M*. *bryophytorum*, the type strains of “*M*. *cellulosiformans*” and *M*. *amethystogenes*, and the type strains of *M*. *triticiradicis* and “*M*. *tritici*”. These observations motivated us to investigate the taxonomy of the genus with whole genome sequences that provide more robust phylogenies and greater discriminatory power.

### Genome based phylogenomic analyses and sequence comparisons

**Fig 1:**
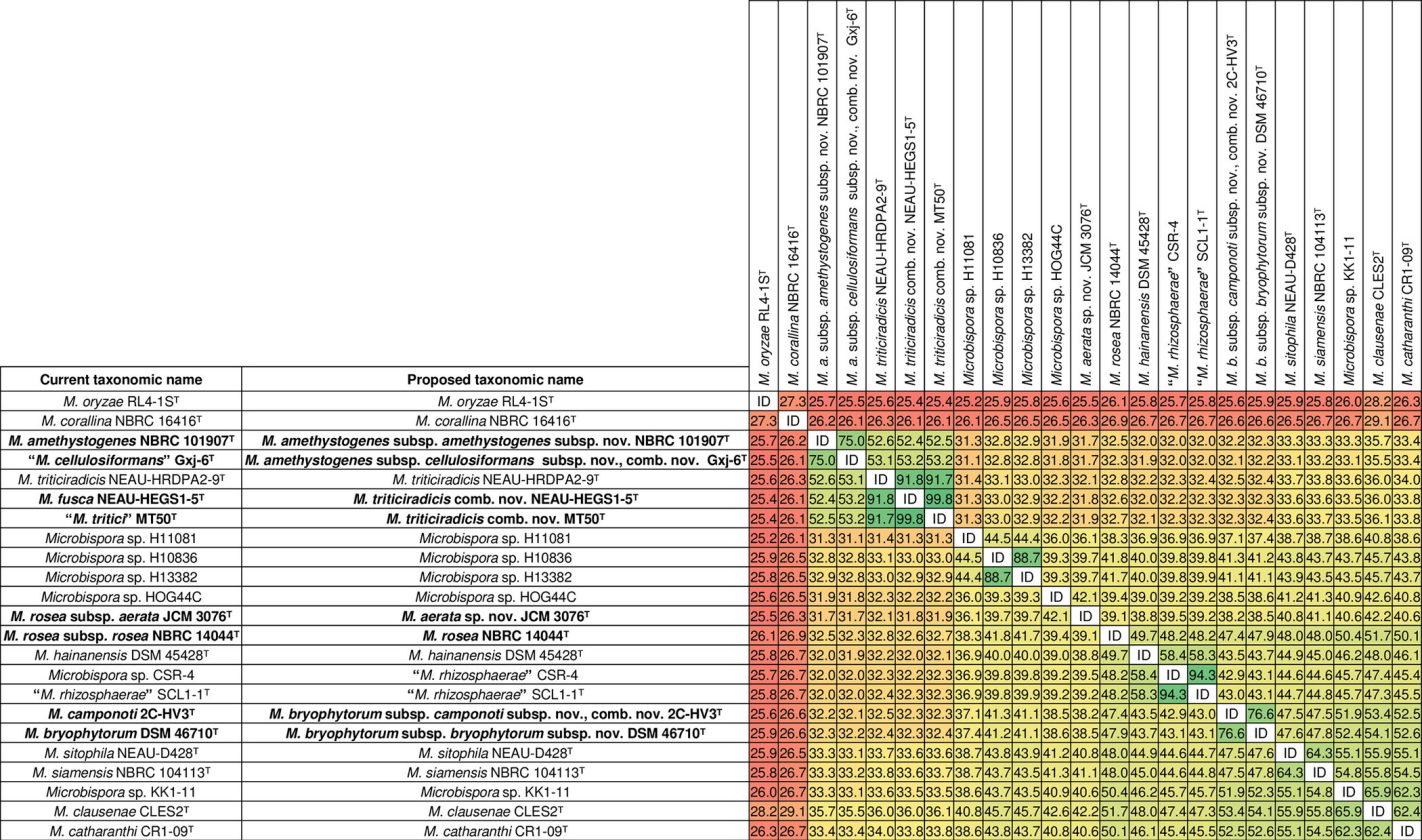
Pairwise comparison of digital DNA-DNA Hybridization (dDDH) values (%) of *Microbispora* strains. Accession numbers of gene sequences used are shown in S1 Table.

**Fig 2:**
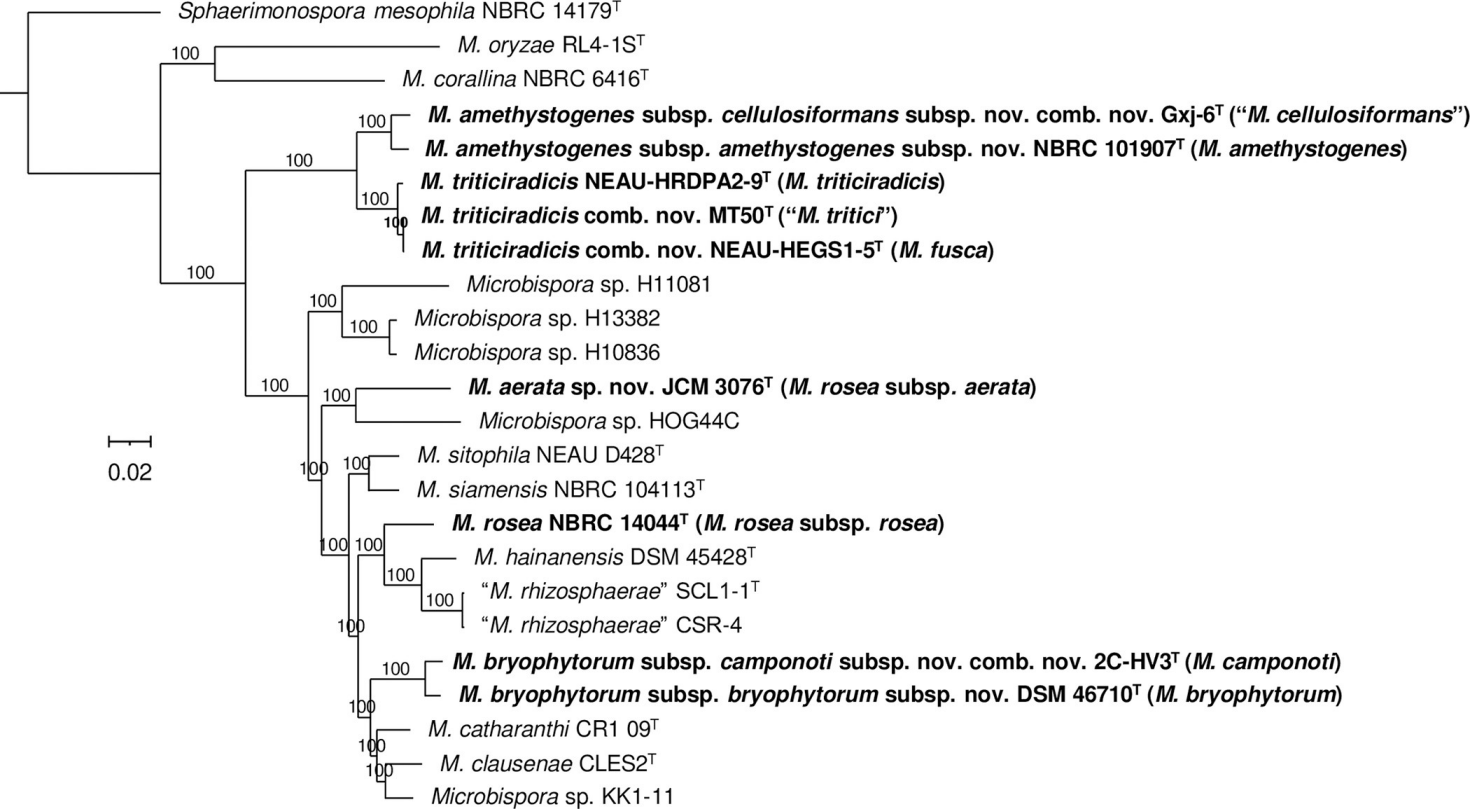
Phylogenetic reconstruction based on core genome sequences of *Microbispora* species. A total of 2625969 nucleotide positions (2443 core genes) were used in the analyzes. Numbers at the nodes represent SH-like branch supports. Bar represents 0.02 nucleotide substitutions per sequence position. Accession numbers of the genome sequences used for the reconstruction are shown in S1 Table.

To clarify the taxonomy of the genus *Microbispora*, we reconstructed phylogenetic relationships using whole-genome sequences of all the 13 species with validly published and correct names (S1 Table). In addition, we included three species “*M*. *cellulosiformans*”, “*M*. *rhizosphaerae*”, and “*M*. *tritici”* with not yet validated names, one synonymized species, *M*. *amethystogenes*, and several additional undescribed *Microbispora* species with public genome sequences. We observed a clear phylogenetic separation between most of the currently accepted species (Fig 1 and S3 Fig). However, according to the tree topology and the sequences similarity values, some of them should be synonymized, and some of the synonymized species represent independent species (Figs 1 and 2, S3 and S4 Figs) as follows. First, the dDDH values between *M*. *amethystogenes* NBRC 101907^T^ and “*M*. *cellulosiformans*” Gxj-6^T^ are 75%, indicating that they represent different subspecies of the same species. We therefore propose to reinstate *M*. *amethystogenes* as an independent species and not as a *M*. *rosea* synonym, reclassify “*M*. *cellulosiformans*” as a subspecies within *M*. *amethystogenes*, and create the following two subspecies: *Microbispora amethystogenes* subsp. *amethystogenes* subsp. nov. with NBRC 101907^T^ (= DSM 43164^T^ = JCM 3021^T^ = NRRL B-2637^T^) as the type strain, and *Microbispora amethystogenes* subsp. *cellulosiformans* subsp. nov., comb. nov. with Gxj-6^T^ (= DSM 109712^T^ = CGMCC 4.7605^T^) as the type strain. Second, we observed that the dDDH values between *M*. *triticiradicis* NEAU-HRDPA2-9^T^, *M*. *fusca* NEAU-HEGS1-5^T^, and “*M*. *tritici*” MT50^T^ are above 90%, indicating that they three belong to the same species. We therefore propose *M*. *fusca* NEAU-HEGS1-5^T^ and “*M*. *tritici*” MT50^T^ as later homotypic synonyms of *M*. *triticiradicis* NEAU-HRDPA2-9^T^. Third, the dDDH value between *M*. *rosea* subsp. *aerata* JCM 3076^T^ and *M*. *rosea* subsp. *rosea* NBRC 14044^T^ is 39.1%, indicating that they belong to different species. We therefore propose to elevate *Microbispora rosea* subsp. *rosea* and *Microbispora rosea* subsp. *aerata* to the species level, and therefore to create *Microbispora aerata* sp. nov. with JCM 3076^T^ (= DSM 43176^T^ = ATCC 15448^T^ = IFO 14624^T^ = NBRC 14624^T^ = VKM Ac-1507^T^) as the type strain. Hence, with this proposition, the correct name for *Microbispora rosea* subsp. *rosea* is *M*. *rosea*. Fourth, the dDDH value between *M*. *camponoti* 2C-HV3^T^ and *M*. *bryophytorum* DSM 46710^T^ is 76.6%, indicating that they represent different subspecies of the same species. We therefore propose to reclassify *M*. *camponoti* as a subspecies within *M*. *bryophytorum*. Consequently, we propose the creation of the following two subspecies: *Microbispora bryophytorum* subsp. *bryophytorum* subsp. nov. with DSM 46710^T^ (= CGMCC 4.7138^T^ = NEAU TX2-2^T^) as the type strain, and *Microbispora bryophytorum* subsp. *camponoti* subsp. nov., comb. nov. with DSM 2C-HV3^T^ (= DSM 100527^T^ = CGMCC 4.7281^T^) as the type strain. All the proposed taxonomic changes are supported also by average nucleotide identity (ANI) values (S4 Fig).

### Taxonomic conclusions

Based on the results of this study, we propose: i) to elevate *Microbispora rosea* subsp. *rosea* and *Microbispora rosea* subsp. *aerata* to the species level, and therefore to create *Microbispora aerata* sp. nov.; ii) to reclassify *M*. *camponoti* as a subspecies within *M*. *bryophytorum*, and therefore to create *Microbispora bryophytorum* subsp. *bryophytorum* subsp. nov. and *Microbispora bryophytorum* subsp. *camponoti* subsp. nov., comb. nov.; iii) to reinstate *M*. *amethystogenes* as an independent species; iv) to reclassify “*M*. *cellulosiformans*” as a subspecies within *M*. *amethystogenes*, therefore to create *Microbispora amethystogenes* subsp. *cellulosiformans* subsp. nov., comb. nov. and *Microbispora amethystogenes* subsp. *amethystogenes* subsp. nov.; and v) to reclassify *M*. *fusca* NEAU-HEGS1-5^T^ and “*M*. *tritici*” MT50^T^ as later homotypic synonyms of *M*. *triticiradicis* NEAU-HRDPA2-9^T^. Future studies should include whole genome sequences and their associated analyses for novel species and subspecies descriptions.

### Future directions

Future research on *Microbispora* species holds immense promise across various disciplines, including biotechnology, medicine, environmental science, and agriculture. In biotechnology, potential applications include the production of cellulases [17], thermophilic and acidophilic chitinases [46], and acetylcholine esterase inhibitors [47]. By exploring these directions, researchers can unlock new bioactive compounds, enhance biotechnological applications, and deepen our understanding of the ecological roles and potential contributions of *Microbispora* species to human health and sustainability. These future research directions highlight the diverse and expanding opportunities to explore the capabilities of *Microbispora* species in advancing science and technology.

### Protologues

#### Description of *Microbispora aerata* sp. Nov

(ae.ra.ta. L. fem. part. adj. *aerata*, covered with bronze). As *Microbispora aerata* sp. nov. results from our proposal to elevate *Microbispora rosea* subsp. *aerata* (Gerber and Lechevalier 1964) to the species level, the description follows Gerber et al. [16] with the following additions. The G+C content of the type-strain genome is 71.46% and its approximate size is 6.8 Mbp, its GenBank deposit GCA_014647835. The type strain is JCM 3076^T^ = DSM 43176^T^ = ATCC 15448^T^ = IFO 14624^T^ = NBRC 14624^T^ = VKM Ac-1507^T^.

#### Emended description of *Microbispora amethystogenes* Nonomura and Ohara, 1960

(a.me.thys.to.ge.nes. L. masc. adj. *amethystinus*, violet colored, amethyst colored; Gr. suff. -genes, producing; from Gr. ind. v. *gennaô*, to produce; N.L. part. adj. *amethystogenes*, producing violet-colored crystals). As this species is proposed to represent an independent species rather than a synonym of *M*. *rosea*, and given that we propose to reclassify “*M*. *cellulosiformans*” as a subspecies of this species, the description follows Nonomura and Ohara [48], Miyadoh et al. [15], Li et al. [4], Han et al. [14], Han et al. [19], Kaewkla et al. [7] and Zhao et al. [9], with the following additions. Growth at pH 5 and 9, at temperatures of 18°C and 40°C, and in the presence of 3% NaCl. This species was found to be positive for gelatin liquefaction and negative for peptonization (coagulation) of skim milk, hydrolysis of starch, decomposition of urea, and production of H_2_S. It exhibited variable results for reduction of nitrate, decomposition of cellulose, and production of catalase. Utilizes lactose and D-mannose as carbon sources, but not L-arabinose, D-galactose, or D-sorbitol. However, this species shows variability for D-xylose, D-fructose, D-mannitol, D-ribose, D-maltose, inositol, D-raffinose, L-rhamnose, and D-sucrose. The tests were positive for L-glutamine and L-serine, but negative for L-tyrosine and creatinine as nitrogen sources. However, the use of L-alanine, L-arginine, L-proline, and L-threonine shows variability. The type strain of the species is NBRC 101907^T^ = DSM 43164^T^ = JCM 3021^T^ = NRRL B-2637^T^. The G+C content of the type-strain genome is 71.61% and its approximate size is 8.1 Mbp. The GenBank accession number is GCA_016863015.1.

#### Description of *Microbispora amethystogenes* subsp. *amethystogenes* subsp. Nov

(a.me.thys.to.ge.nes. L. masc. adj. *amethystinus*, violet colored, amethyst colored; Gr. suff. *-genes*, producing; from Gr. ind. v. *gennaô*, to produce; N.L. part. adj. *amethystogenes*, producing violet-colored crystals). This taxon results for the proposition to reclassify “*Microbispora cellulosiformans*” as a subspecies of *Microbispora amethystogenes*. This proposition automatically creates *Microbispora amethystogenes* subsp. *amethystogenes* subsp. nov. Therefore, the description is as given for *Microbispora amethystogenes* [48], with the following additions. The G+C content of the type-strain genome is 71.61% and its approximate size is 8.1 Mbp, its GenBank deposit GCA_016863015. The type strain is NBRC 101907^T^ = DSM 43164^T^ = JCM 3021^T^ = NRRL B-2637^T^.

#### Description of *Microbispora amethystogenes* subsp. *cellulosiformans* subsp. Nov

(cel.lu.lo.si.for”mans. N.L. neut. n. *cellulosum*, cellulose; L. pres. part. *formans*, forming; N.L. part. adj. *cellulosiformans*, producing cellulose). This taxon results for the proposition to reclassify “*Microbispora cellulosiformans*” as a subspecies of *Microbispora amethystogenes*. Therefore, the description is as given for “*Microbispora cellulosiformans*” [17] with the following additions. The G+C content of the type-strain genome is 71.58% and its approximate size is 8.4 Mbp. The GenBank accession number is GCA_008728085. The type strain is Gxj-6^T^ = DSM 109712^T^ = CGMCC 4.7605^T^.

#### Emended description of *Microbispora bryophytorum* Li et al. 2015

(bry.o.phy.to”rum. N.L. gen. neut. pl. n. *bryophytorum* pertaining to the botanical phylum *Bryophyta*). This emendation is the result of our proposition to reclassify *Microbispora camponoti* as *Microbispora bryophytorum* subsp. *camponoti* subsp. nov. comb. nov., and to create *Microbispora bryophytorum* subsp. *bryophytorum* subsp. nov. Hence, the description of this species follows Li et al. [4], with the following additions. Variable for starch hydrolysis, gelatin liquefaction, urea hydrolysis, utilization of L-arabinose, *meso*-inositol and D-ribose as sole carbon sources, and utilization of L-proline and L-glutamine as sole nitrogen source. The G+C content of the type-strain genome is 71.13% and its approximate size is 7.8 Mbp. The GenBank accession number of the genome is GCA_006874465. The type strain is DSM 46710^T^ = CGMCC 4.7138^T^ = NEAU TX2-2^T^.

#### Description of *Microbispora bryophytorum* subsp. *bryophytorum* subsp. Nov

(bry.o.phy.to”rum. N.L. gen. neut. pl. n. *bryophytorum* pertaining to the botanical phylum *Bryophyta*). Following our proposition to reclassify *Microbispora camponoti* as *Microbispora bryophytorum* subsp. *camponoti* subsp. nov. comb. nov., and to create *Microbispora bryophytorum* subsp. *bryophytorum* subsp. nov., the description of this subspecies follows Li et al. [4], with the following additions. The G+C content of the type-strain genome is 71.13% and its approximate size is 7.8 Mbp. The GenBank accession number is GCA_006874465. The type strain is DSM 46710^T^ = CGMCC 4.7138^T^ = NEAU TX2-2^T^.

#### Description of *Microbispora bryophytorum* subsp. *camponoti* subsp. Nov

(cam.po.no”ti. N.L. gen. masc. n. *camponoti*, of Camponotus, referring to the insect *Camponotus japonicus* Mayr from which the type strain was isolated). Following our proposition to reclassify *Microbispora camponoti* as *Microbispora bryophytorum* subsp. *camponoti* subsp. nov. comb. nov., the description of this subspecies is identical to the description given by Han et al. [5], with the following additions. The G+C content of the type-strain genome is 71.01% and its approximate size is 8.0 Mbp. The GenBank accession number is GCA_014712745. The type strain is 2C-HV3^T^ = DSM 100527^T^ = CGMCC 4.7281^T^.

#### Emended description of *Microbispora rosea* Nonomura and Ohara 1957

(ro.se”a. L. fem. adj. *rosea*, rose colored). As we propose to elevate *Microbispora rosea* subsp. *rosea* Nonomura and Ohara 1957 to the species level, the description of *Microbispora rosea* follows Nonomura and Ohara [1] with the following additions. The G+C content of the type-strain genome is 71.17% and its approximate size is 8.8 Mbp. The GenBank accession number is GCA_016863055. The type strain is NBRC 14044^T^ = DSM 43839^T^ = ATCC 12950^T^ = IFO 14044^T^ = JCM 3006^T^ = NRRL B-2632^T^ = VKM Ac-634^T^.

#### Emended description of *Microbispora triticiradicis*

(tri.ti.ci.ra”di.cis. L. neut. n. *triticum*, wheat; L. fem. n. *radix*, a root; N.L. gen. fem. n. *triticiradicis*, of a wheat root, referring to the isolation of the organism from root of *Triticum aestivum* L.). Basonyms: “*Microbispora tritici*” Han et al. 2019 and *M*. *fusca* Zhao et al. 2020. Since we propose “*Microbispora tritici*” and *M*. *fusca* as later homotypic synonyms of this species, the description is based on Han et al. [19], Gong et al. [17], Klykleung et al. [6], and Zhao et al. [9], with the following additions. *M*. *triticiradicis* grows at pH levels of 5 and 8, at a temperature of 45°C, and in the presence of 2% NaCl. This species was positive for the degradation of aesculin and the utilization of D-glucose, D-fructose, raffinose, D-sorbitol, and D-sucrose as sole carbon sources. It also showed the ability to utilize L-alanine, L-arginine, L-asparagine, L-aspartic acid, L-glutamic acid, L-glutamine, and L-proline as sole nitrogen sources. This species, however, tested negative for the degradation of Tweens 20 and 80, liquefaction of gelatin, peptonization of skim milk, reduction of nitrate, and production of H_2_S. Additionally, it is unable to use L-arabinose, inositol, L-rhamnose, D-ribose, and D-xylose as sole carbon sources. Moreover, it cannot utilize glycine and L-tyrosine as sole nitrogen sources. However, this species showed variability in the decomposition of cellulose, Tween 40, urea, hydrolysis of starch, production of catalase, utilization of lactose, D-mannitol, maltose, D-mannose, and D-galactose as sole carbon sources; as well as the utilization of L-threonine, creatine, and L-serine as sole nitrogen sources. The G+C content of the type-strain genome is 71.68% and its approximate size is 8 Mbp. The GenBank accession number is GCA_003260025. The type strain is NEAU-HRDPA2-9^T^ = DSM 104649^T^ = CGMCC 4.7399^T^.

## Supporting information

S1 Fig Pairwise comparison of 16S rRNA values (%) of *Microbispora* strains. Accession numbers of gene sequences used are shown in the phylogenetic tree of S2 Fig and S2 Table.
(PPT)

S2 Fig Maximum-likelihood phylogenetic tree reconstructed from 16S rRNA gene sequences of all *Microbispora* child taxa. The evolutionary history was inferred by using the maximum-likelihood method based on the Tamura-Nei model. The tree with the highest log likelihood is shown. The percentage of trees in which the associated taxa clustered together is shown next to the branches. The tree is drawn to scale, with branch lengths measured in the number of substitutions per site. There were a total of 1349 positions in the final dataset. Evolutionary analyses were conducted in MEGA7 based on 100 replications. Accession numbers of gene sequences used are shown in parenthesis.
(PPT)

S3 Fig Whole-genome based phylogenetic tree of *Microbispora*. Trees were inferred with FastME 2.1.6.1 from GBDP distances calculated from genome sequences. The branch lengths are scaled in terms of GBDP distance formula *d*_*5*_. The numbers above branches are GBDP pseudo-bootstrap support values from 100 replications. Scientific names shown in parenthesis correspond to the current scientific names. Names in bold are to indicate the proposed taxonomic changes. NCBI accession numbers of the sequences used for the analyses are shown in S1 Table.
(PPT)

S4 Fig Pairwise comparison of Average Nucleotide Identity (ANI) values (%) of *Microbispora* strains. Accession numbers of gene sequences used are shown in S1 Table. Scientific names used correspond to the proposed taxonomic names instead of the current scientific names.
(PPT)

S1 Table Features of the genome sequences used in this study. (PDF)

S2 Table Characteristics of the 16S rRNA gene sequences used in this study. (PDF)
