# Supplementary material for: Whole genome-based reclassification of several species of the genus Microbispora
Source: PLoS One. 2024 Aug 22;19(8):e0307299. doi: 10.1371/journal.pone.0307299 (PMC11341043; doi:10.1371/journal.pone.0307299)
Supplement: S1 Fig — Accession numbers of gene sequences used are shown in the phylogenetic tree of S2 Fig and S2 Table. (PPT) [file pone.0307299.s001.ppt]

## Slide 1
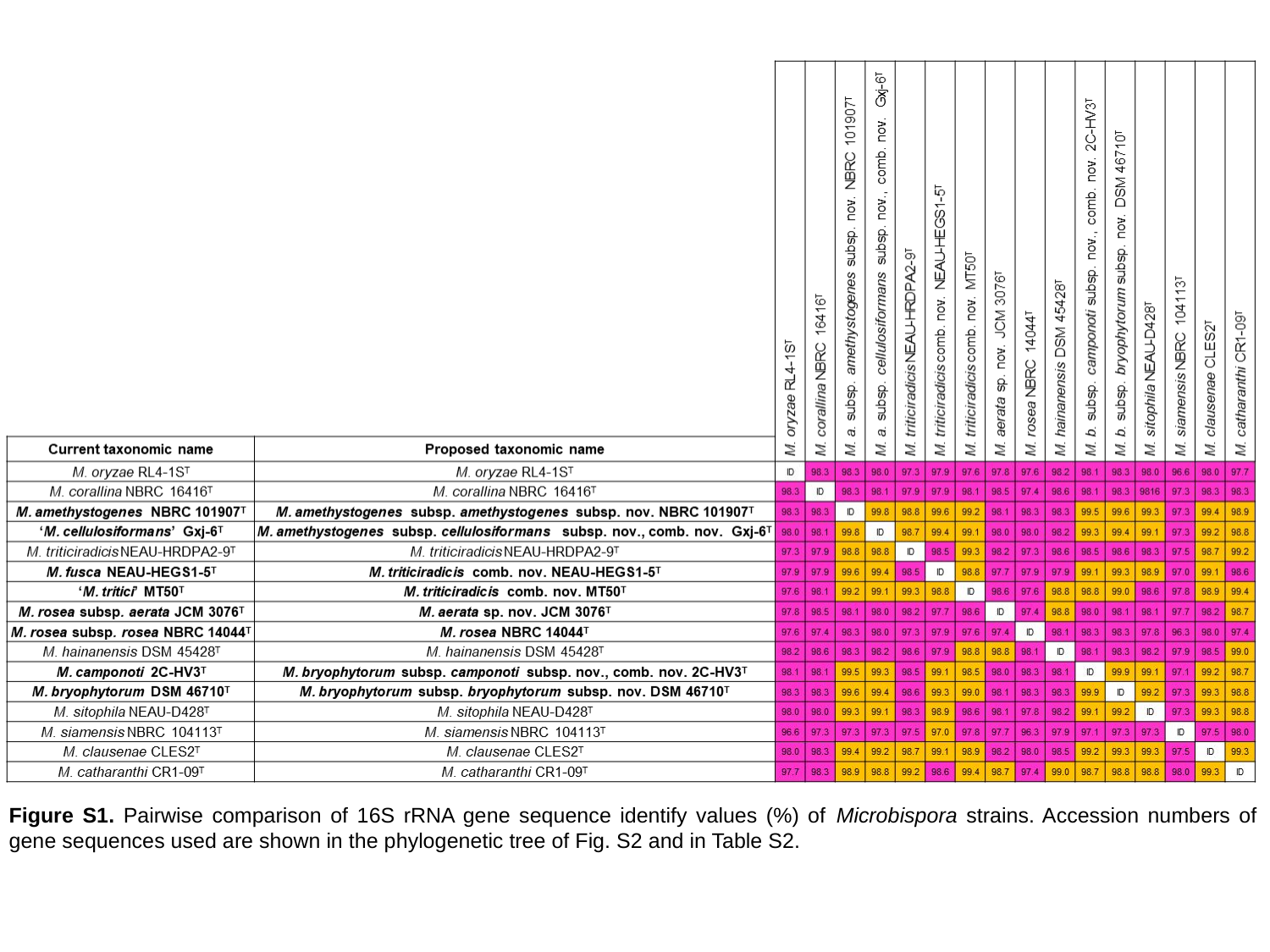

Figure S1. Pairwise comparison of 16S rRNA gene sequence identify values (%) of Microbispora strains. Accession numbers of gene sequences used are shown in the phylogenetic tree of Fig. S2 and in Table S2.
