# Supplementary material for: Whole genome-based reclassification of several species of the genus Microbispora
Source: PLoS One. 2024 Aug 22;19(8):e0307299. doi: 10.1371/journal.pone.0307299 (PMC11341043; doi:10.1371/journal.pone.0307299)
Supplement: S3 Fig — Trees were inferred with FastME 2.1.6.1 from GBDP distances calculated from genome sequences. The branch lengths are scaled in terms of GBDP distance formula d5. The numbers above branches are GBDP pseudo-bootstrap support values from 100 replications. Scientific names shown in parenthesis correspond to the current scientific names. Names in bold are to indicate the proposed taxonomic changes. NCBI accession numbers of the sequences used for the analyses are shown in S1 Table. (PPT) [file pone.0307299.s003.ppt]

## Slide 1
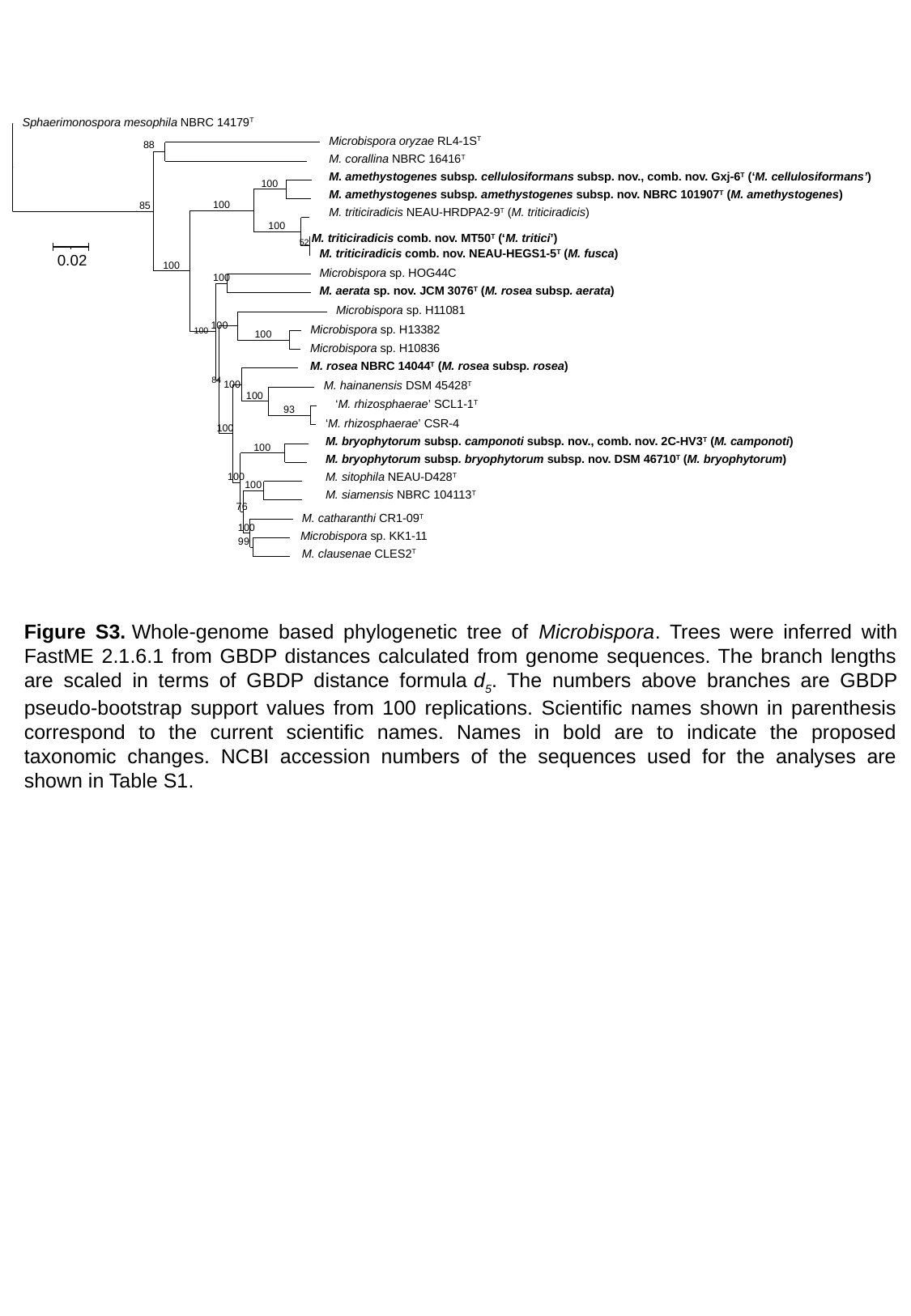

Sphaerimonospora mesophila NBRC 14179T
88
Microbispora oryzae RL4-1ST
M. corallina NBRC 16416T
M. amethystogenes subsp. cellulosiformans subsp. nov., comb. nov. Gxj-6T (‘M. cellulosiformans’)
M. amethystogenes subsp. amethystogenes subsp. nov. NBRC 101907T (M. amethystogenes)
M. triticiradicis NEAU-HRDPA2-9T (M. triticiradicis)
100
100
85
100
52 M. triticiradicis comb. nov. MT50T (‘M. tritici’)
M. triticiradicis comb. nov. NEAU-HEGS1-5T (M. fusca)
Microbispora sp. HOG44C
M. aerata sp. nov. JCM 3076T (M. rosea subsp. aerata)
0.02
100
100
Microbispora sp. H11081
100 100
Microbispora sp. H13382
100
Microbispora sp. H10836
M. rosea NBRC 14044T (M. rosea subsp. rosea)
84 100
M. hainanensis DSM 45428T
100
‘M. rhizosphaerae’ SCL1-1T
93
‘M. rhizosphaerae’ CSR-4
M. bryophytorum subsp. camponoti subsp. nov., comb. nov. 2C-HV3T (M. camponoti)
M. bryophytorum subsp. bryophytorum subsp. nov. DSM 46710T (M. bryophytorum)
M. sitophila NEAU-D428T
M. siamensis NBRC 104113T
100
100
100
100
76
M. catharanthi CR1-09T Microbispora sp. KK1-11
M. clausenae CLES2T
100
99
Figure S3. Whole-genome based phylogenetic tree of Microbispora. Trees were inferred with FastME 2.1.6.1 from GBDP distances calculated from genome sequences. The branch lengths are scaled in terms of GBDP distance formula d5. The numbers above branches are GBDP pseudo-bootstrap support values from 100 replications. Scientific names shown in parenthesis correspond to the current scientific names. Names in bold are to indicate the proposed taxonomic changes. NCBI accession numbers of the sequences used for the analyses are shown in Table S1.
