# Supplementary material for: Whole genome-based reclassification of several species of the genus Microbispora
Source: PLoS One. 2024 Aug 22;19(8):e0307299. doi: 10.1371/journal.pone.0307299 (PMC11341043; doi:10.1371/journal.pone.0307299)
Supplement: S4 Fig — Accession numbers of gene sequences used are shown in S1 Table. Scientific names used correspond to the proposed taxonomic names instead of the current scientific names. (PPT) [file pone.0307299.s004.ppt]

## Slide 1
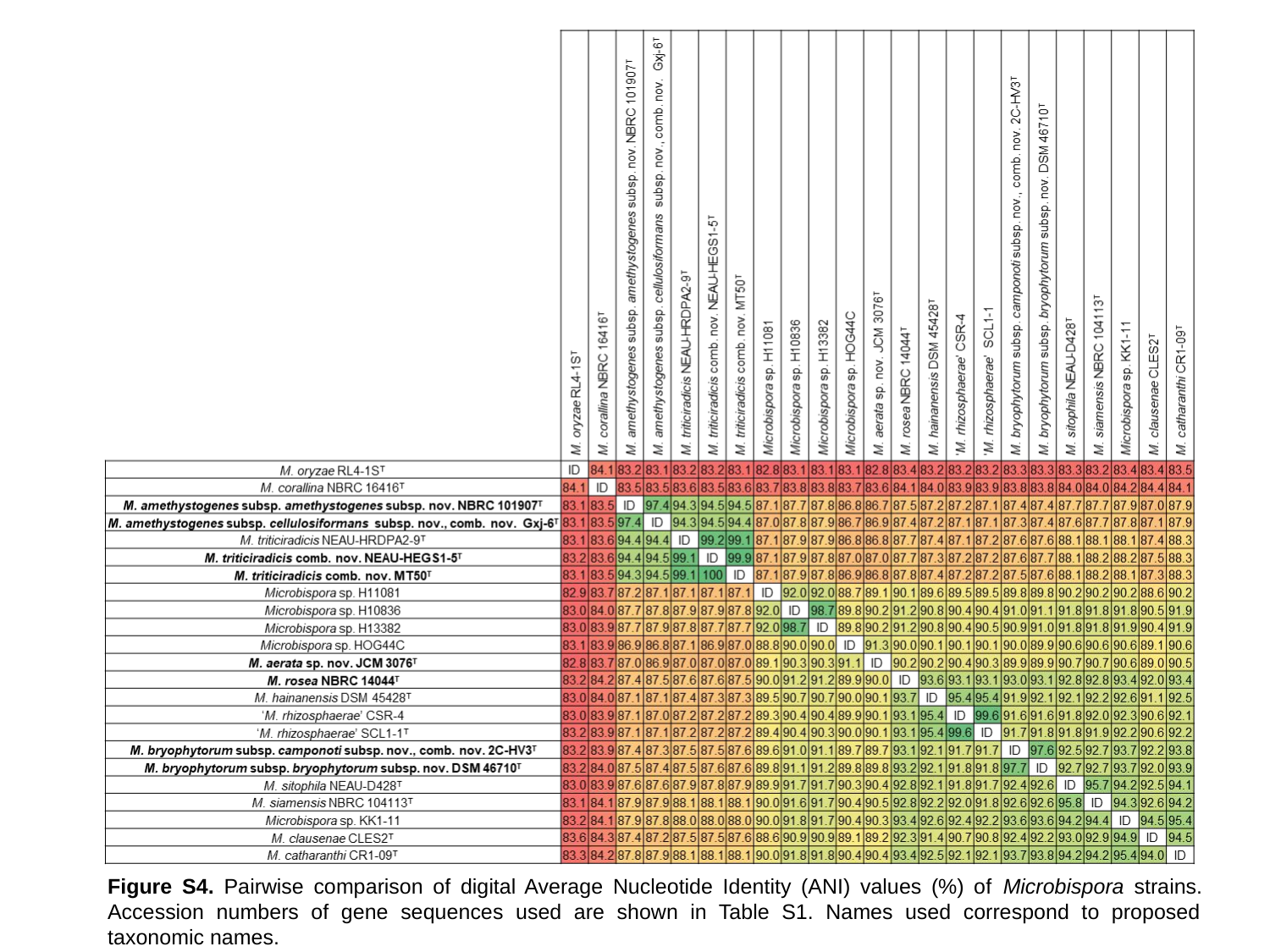

Figure S4. Pairwise comparison of digital Average Nucleotide Identity (ANI) values (%) of Microbispora strains. Accession numbers of gene sequences used are shown in Table S1. Names used correspond to proposed taxonomic names.
