# Supplementary material for: Whole genome-based reclassification of several species of the genus Microbispora
Source: PLoS One. 2024 Aug 22;19(8):e0307299. doi: 10.1371/journal.pone.0307299 (PMC11341043; doi:10.1371/journal.pone.0307299)
Supplement: S1 Table — (PDF) [file pone.0307299.s005.pdf]

Table S1. Features of the genome sequences used in this study.

Current taxonomic name	Proposed taxonomic name	Genome assembly	GenBank assembly accession number	Total length (bp)	GC content (%)	Number of proteins	Completeness (%)	Contamination (%)
***M. amethystogenes* NBRC 101907ᵀ**	***M. amethystogenes* subsp. *amethystogenes* subsp. nov. NBRC 101907ᵀ**	ASM1686301v1	GCA_016863015.1	8,135,544	71.61	7209	100	0.8
***M. bryophytorum* DSM 46710ᵀ**	***M. bryophytorum* subsp. *bryophytorum* subsp. nov. DSM 46710ᵀ**	ASM687446v1	GCA_006874465.1	7,853,874	71.13	7209	99.9	1.1
***M. camponoti* 2C-HV3ᵀ**	***M. bryophytorum* subsp. *camponoti* subsp. nov., comb. nov. 2C-HV3ᵀ**	ASM1471274v1	GCA_014712745.1	8,015,934	71.01	7347	100	0.7
M. catharanthi CR1-09ᵀ	*M. catharanthi* CR1-09ᵀ	ASM633491v2	GCA_006334915.2	9,251,614	71.22	8510	99.8	1.4
**'*M. cellulosiformans*' Gxj-6ᵀ**	***M. amethystogenes* subsp. *cellulosiformans* subsp. nov., comb. nov. Gxj-6ᵀ**	ASM872808v1	GCA_008728085.1	8,445,470	71.58	7362	100	0.9
M. clausenae CLES2ᵀ	*M. clausenae* CLES2ᵀ	ASM1387060v1	GCA_013870605.1	7,257,597	69.97	8717	83.4	2.2
M. corallina NBRC 16416ᵀ	*M. corallina* NBRC 16416ᵀ	ASM1686303v1	GCA_016863035.1	8,361,641	72.36	7749	100	1.7
***M. fusca* NEAU-HEGS1-5ᵀ**	***M. triticiradicis* comb. nov. NEAU-HEGS1-5ᵀ**	ASM586406v1	GCA_005864065.1	8,152,858	71.68	7063	99.3	1.2
M. hainanensis DSM 45428ᵀ	*M. hainanensis* DSM 45428ᵀ	ASM687447v1	GCA_006874475.1	8,717,787	71.25	8095	100	1.0
M. oryzae RL4-1Sᵀ	*M. oryzae* RL4-1Sᵀ	ASM1789616v1	GCA_017896165.1	7,464,125	71.21	6632	100	2.2
***M. rosea* subsp. *aerata* JCM 3076ᵀ**	***M. aerata* sp. nov. JCM 3076ᵀ**	ASM1464783v1	GCA_014647835.1	6,884,475	71.46	6130	100	1.9
***M. rosea* subsp. *rosea* NBRC 14044ᵀ**	***M. rosea* NBRC 14044ᵀ**	ASM1686305v1	GCA_016863055.1	8,827,503	71.17	8209	99.9	0.9
M. siamensis NBRC 104113ᵀ	*M. siamensis* NBRC 104113ᵀ	ASM1686309v1	GCA_016863095.1	9,393,388	71.42	8561	99.8	0.3
M. sitophila NEAU-D428ᵀ	*M. sitophila* NEAU-D428ᵀ	ASM1489224v1	GCA_014892245.1	10,088,235	71.19	9117	100	1.5
**'*M. tritici*' MT50ᵀ**	***M. triticiradicis* comb. nov. MT50ᵀ**	ASM808599v1	GCA_008085995.1	8,039,471	71.72	7112	100	1.2
M. triticiradicis NEAU-HRDPA2-9ᵀ	*M. triticiradicis* NEAU-HRDPA2-9ᵀ	ASM326002v2	GCA_003260025.2	8,096,094	71.68	7251	100	4.0
Microbispora sp. CSR-4	*Microbispora* sp. CSR-4	ASM803368v1	GCA_008033685.1	8,666,412	71.28	8061	100	1.7
Microbispora sp. H10836	*Microbispora* sp. H10836	ASM1299952v1	GCA_012999525.1	8,828,565	71.32	8074	100	1.1
Microbispora sp. H11081	*Microbispora* sp. H11081	ASM1299953v1	GCA_012999535.1	7,378,305	71.46	6643	100	0.7
Microbispora sp. H13382	*Microbispora* sp. H13382	ASM1416144v1	GCA_014161445.1	8,711,424	71.4	7876	100	0.6
Microbispora sp. HOG44C	*Microbispora* sp. HOG44C	ASM1968965v1	GCA_019689655.1	6,336,961	71.31	6332	97.9	1.5
Microbispora sp. KK1-11	*Microbispora* sp. KK1-11	ASM687443v1	GCA_006874435.1	9,295,408	71.33	8655	100	0.7
'*M. rhizosphaerae*' SCL1-1ᵀ	'*M. rhizosphaerae*' SCL1-1ᵀ	ASM687445v1	GCA_006874455.1	8,812,224	71.24	8159	99.8	1.4
Sphaerimonospora mesophila NBRC 14179ᵀ	*Sphaerimonospora mesophila* NBRC 14179ᵀ	ASM131368v1	GCA_001313685.1	4,823,032	67.79	9384	99.0	1.2
