# Supplementary material for: Whole genome-based reclassification of several species of the genus Microbispora
Source: PLoS One. 2024 Aug 22;19(8):e0307299. doi: 10.1371/journal.pone.0307299 (PMC11341043; doi:10.1371/journal.pone.0307299)
Supplement: S2 Table — (PDF) [file pone.0307299.s006.pdf]

Table S2 Characteristics of the 16S rRNA gene sequences used in this study

Current taxonomic name	Proposed taxonomic name	Accession number	Length (pb)	Differences (in nucleotides or gaps) between the 16S rRNA sequences from the PCR and the whole genome assembly/% blast
***M. amethystogenes* NBRC 101907^T**	***M. amethystogenes* subsp. *amethystogenes* subsp. nov. NBRC 101907^T**	U48988	1413	11/1413, 99.22%
***M. bryophytorum* DSM 46710^T**	***M. bryophytorum* subsp. *bryophytorum* subsp. nov. DSM 46710^T**	KF886293	1513	1/1513, 99.93%
***M. camponoti* 2C-HV3^T**	***M. bryophytorum* subsp. *camponoti* subsp. nov., comb. nov. 2C-HV3^T**	KR261651	1513	1/1513, 99.93%
M. catharanthi CR1-09^T	*M. catharanthi* CR1-09^T	LC082231	1469	0/1469, 100%
**‘*M. cellulosiformans*’ Gxj-6^T**	***M. amethystogenes* subsp. *cellulosiformans* subsp. nov., comb. nov. Gxj-6^T**	MK422546	1513	3/1513, 99.80%
M. clausenae CLES2^T	*M. clausenae* CLES2^T	KX394342	1452	0/1452, 100%
M. corallina NBRC 16416^T	*M. corallina* NBRC 16416^T	AB018046	1442	8/1442, 99.45%
***M. fusca* NEAU-HEGS1-5^T**	***M. triticiradicis* comb. nov. NEAU-HEGS1-5^T**	MK825340	1514	0/1514, 100%
M. hainanensis DSM 45428^T	*M. hainanensis* DSM 45428^T	FJ261972	1480	3/1480, 99.86%
M. oryzae RL4-1S^T	*M. oryzae* RL4-1S^T	MW559531	1417	0/1417, 100%
***M. rosea* subsp. *aerata* JCM 3076^T**	***M. aerata* sp. nov. JCM 3076^T**	MT760396	1297	5/1297, 99.61%
***M. rosea* subsp. *rosea* NBRC 14044^T**	***M. rosea* NBRC 14044^T**	D86936	1473	12/1473, 99.19%
M. siamensis NBRC 104113^T	*M. siamensis* NBRC 104113^T	CC01049601	1470	0/1470, 100%
M. sitophila NEAU-D428^T	*M. sitophila* NEAU-D428^T	MW001208	1521	5/1521, 99.67%
**‘*M. tritici*’ MT50^T**	***M. triticiradicis* comb. nov. MT50^T**	MF969267	1513	2/1513, 99.87%
M. triticiradicis NEAU-HRDPA2-9^T	*M. triticiradicis* NEAU-HRDPA2-9^T	MF969266	1515	4/1515, 99.48%
Microbispora sp. CSR-4	*Microbispora* sp. CSR-4	LC383886	1498	1/1498, 99.93%
Microbispora sp. H10836	*Microbispora* sp. H10836	LC363903	1484	6/1484, 99.80%
Microbispora sp. H11081	*Microbispora* sp. H11081	LC363911	1337	3/1337, 99.78%
Microbispora sp. H13382	*Microbispora* sp. H13382	LC363913	1302	0/1302, 100%
Microbispora sp. KK1-11	*Microbispora* sp. KK1-11	LC333389	1480	6/1480, 99.59%
‘*M. rhizosphaerae*’ SCL1-1^T	‘*M. rhizosphaerae*’ SCL1-1^T	OM442437	1521	0/1521, 100%
Sphaerimonospora mesophila NBRC 14179^T	*Sphaerimonospora mesophila* NBRC 14179^T	AF002266	1456	2/1456, 99.8%
